# Seroprevalence and Risk Factors of *Chlamydia* Infection in Domestic Rabbits (*Oryctolagus cuniculus*) in China

**DOI:** 10.1155/2015/460473

**Published:** 2015-04-05

**Authors:** Xiaoting Ni, Siyuan Qin, Zhilong Lou, Hongrui Ning, Xiaolin Sun

**Affiliations:** ^1^College of Veterinary Medicine, Gansu Agricultural University, Lanzhou, Gansu 730070, China; ^2^State Key Laboratory of Veterinary Etiological Biology, Lanzhou Veterinary Research Institute, Chinese Academy of Agricultural Sciences, Lanzhou, Gansu 730046, China; ^3^College of Animal Science and Technology, Jilin Agriculture University, Changchun, Jilin 130118, China; ^4^College of Animal Science and Technology, Anhui Agricultural University, Hefei, Anhui 230000, China

## Abstract

*Chlamydia* spp. are obligate intracellular bacteria distributed all over the world, known to cause various forms of diseases in animals and humans. In the present study, a serological survey was conducted to detect the seroprevalence and risk factors associated with rabbit chlamydiosis in northeast China, including Liaoning province, Jilin province, Heilongjiang province, and Inner Mongolia Autonomous Region. Antibodies to *Chlamydia* were determined by indirect hemagglutination assay (IHA). The overall seroprevalence was estimated at 17.88% in total of 800 blood samples. The *Chlamydia* seroprevalence varied in domestic rabbits from different factors, and genders of domestic rabbits were considered as major risk factors associated with *Chlamydia* infection. Our study revealed a widespread and high prevalence of *Chlamydia* infection in domestic rabbits in northeast China, with higher exposure risk in female domestic rabbits. These findings suggested the potential importance of domestic rabbits in the transmission of zoonotic *Chlamydia* infection, and thus *Chlamydia* should be taken into consideration in diagnosing rabbit diseases. To our knowledge, there is no report of *Chlamydia* infection in domestic rabbits in China and the results extend the host range for *Chlamydia*, which has important implications for public health and the local economy.

## 1. Introduction

*Chlamydia* is a genus comprising important zoonotic obligate intracellular pathogens that affect humans and a wide range of animals [1, 2].* Chlamydia* infection can cause various diseases in nonhuman mammals and birds, including conjunctivitis, atypical pneumonia, enteritidis, endocarditis, and even abortion, resulting in serious economic losses [3–6]. Several* Chlamydia* species are known to infect humans and are of serious public health significance because they may result in atherosclerosis, coronary heart disease, pneumonia, and other severe diseases [7].* Chlamydia abortus* and* Chlamydia psittaci* are of particular importance because they can cause abortion and psittacosis, respectively, in animals, birds and humans. In Egypt, a study revealed that in symptomatic gynecologically diseased women the seroprevalence of* Chlamydia psittaci* was 50.0%,* Chlamydia abortus* was 35.7%, and* Chlamydia trachomatis* was 15.2% [8], and in the genital tract of cattle the main Chlamydiaceae were* Chlamydia psittaci* and* Chlamydia abortus* [9]. The animal diseases caused by these microorganisms should be given more attention due to their zoonotic potential [1, 7, 10].

The breeding of domestic rabbits (*Oryctolagus cuniculus*) for human consumption has a long radition in China, and rabbit industry is one of green economy industries, which is emerging and promising, with broad space for development and has gone through a long glorious history and its products have brought great wealth to the rural people in China. At present, China plays an important role of breeding rabbits, and domestic rabbits yield high quality meat and hair which are transported to many parts of home and abroad. However, disease is the main challenge for rabbit industry [11, 12]. To date, there is little information about the seroprevalence of* Chlamydia* and the risk factors associated with* Chlamydia* infection in domestic rabbits in the world. Thus, the present study was aimed at determining the seroprevalence of* Chlamydia* infection in domestic rabbits in northeast China and identifying factors associated with the presence of* Chlamydia* antibodies in domestic rabbits in the study area.

## 2. Materials and Methods

### 2.1. Ethics Statement

This study was approved by the Animal Ethics Committee of Lanzhou Veterinary Research Institute, Chinese Academy of Agricultural Sciences (Approval no. LVRIAEC2013-010). The domestic rabbits, from which the serum samples were collected, were handled in accordance with good animal practices required by the Animal Ethics Procedures and Guidelines of the People's Republic of China.

### 2.2. Collection and Preparation of Serum Samples

A total of eight hundred domestic rabbits were randomly sampled from Liaoning province, Jilin province, Heilongjiang province, and Inner Mongolia Autonomous Region. From each rabbit, approximately 2 mL of blood was collected from the marginal ear vein or postmortem. After centrifugation at 3,000 rpm for 10 min, sera were collected and stored at −20°C until use. Handling of rabbits was in compliance with the Animal Ethics Procedures and Guidelines of the People's Republic of China. Information about species, ages, geographic origin, and gender was acquired from breeders.

### 2.3. Serological Examination

Antibodies to* Chlamydia* were tested by indirect hemagglutination antibody (IHA) using a commercially marketed kit (Lanzhou Veterinary Research Institute, Chinese Academy of Agricultural Sciences, Lanzhou, Gansu Province, China). The procedures are according to the manufacturer's instructions and previous descriptions [13, 14]. The IHA kit was performed following the standard protocol recommended by Chinese Center for Animal Diseases Prevention and Control, and the sensitivity and specificity of the test are 100% and 95%, respectively [13]. The sensitivity and specificity values for the testing kit used in this study have been validated by The Ministry of Agriculture of the People's Republic of China (NY/T 562–2002). In brief, 75 *μ*L of the IHA dilution solution was transferred into 96 well V-bottomed polystyrene plates with 25 *μ*L of serum added and diluted in a fourfold series from 1 : 4 to 1 : 64. The plates were shaken for 2 min and then incubated at 37°C for 2 h without shaking. The test was considered positive when a layer of agglutinated erythrocytes was formed in wells at dilutions of 1 : 16 or higher, and positive and negative controls were included in each test. Those sera which showed dubious results were picked out for retest.

### 2.4. Statistical Analyses

Regions, genders, age groups, and breeds of rabbits were analyzed in a multivariable logistic regression model, and probability (*P*) value < 0.05 was considered as statistically significant. Odds ratios (OR) with 95% confidence intervals are also calculated. All statistical analyses were performed using the PASW Statistics 18.0 (SPSS Inc., IBM Corporation, Somers, NY).

## 3. Results

Antibodies to* Chlamydia* were detected in 143 of 800 (17.88%, 95% CI 15.22–20.53) domestic rabbits. Of these, antibody titers were 1 : 16 in 22, 1 : 32 in 18, 1 : 64 in 33, 1 : 128 in 30, 1 : 256 in 27, 1 : 512 in 9, and 1 : 1024 in 4, respectively (Table 3). The* Chlamydia* seroprevalence varied in domestic rabbits from different regions, ranging from 15.00% (95% CI 10.05–19.95) to 20.50% (95% CI 14.91–26.10) (Table 1), but the differences were not significantly different. The juvenile rabbits had higher seroprevalence (19.83%) than that in adults (15.18%). The seroprevalence of* Chlamydia* was diverse in different breeds of domestic rabbits; the highest level was 20.88% in Chinese White Rabbits, followed by 17.61% in New Zealand Rabbits and 15.20% in California Rabbits (Table 1). However, the differences were not statistically significant (*P* > 0.05).

In terms of gender, seroprevalence of* Chlamydia* in female (20.34%) was higher than that in male (14.11%), and gender was considered as major risk factors associated with* Chlamydia* infection in domestic rabbits (*P* < 0.05, Table 2). And the risk of* Chlamydia* infection in female was more than 1.56-fold increase (OR = 1.56, 95% CI = 1.06–2.29, *P* < 0.024) compared to* Chlamydia* infection in male (Table 2).

## 4. Discussion

*Chlamydia* spp. are gram-negative bacteria that cause various significant diseases in humans and animals all over the world, leading to health problems and serious economic losses. In the present study, the overall seroprevalence of* Chlamydia* infection in domestic rabbits was 17.88%, which was higher than that observed previously in cats (5.9%) [7], dogs (12.1%) [15], pet birds (10.80%) [16], pigeon (6.80%) [17], red deer (9.60%) [18], cattle (4.75%) [19] and dairy cattle (7.25%) [20], but lower than in sows (62.70%) [21], wild boar (63.60%) [22], and feral Canada geese (93.80%) [23] using the same serological test (IHA) in China. Differences in* Chlamydia* seroprevalence are likely due to differences in animal welfares, animal categories, climates, and husbandry practices. Today, there is an increasing demand for rabbit meat in China, and the safety and sanitary quality of meat pose a potential threat to local residents and other people in exported regions. Therefore, it is important to understand the extent of* Chlamydia* infection in domestic rabbits in China.

In the present study,* Chlamydia* seroprevalence in juvenile rabbits (19.83%) was higher than that in adult rabbits (15.18%), but the differences were not statistically significant (*P* > 0.05), which was consistent with the previous study [14]. And the reason why juveniles were more susceptible than adults and subadults may be that the juveniles had lower immunity compared with adults. Moreover, the* Chlamydia* seroprevalence between male and female rabbits was significantly different (*P* < 0.05), implying that gender is a crucial factor for* Chlamydia* infection in domestic rabbits.

This study revealed that gender is a significant risk factor for* Chlamydia* prevalence in domestic rabbits, with females having a higher seroprevalence than males. The* Chlamydia* seroprevalence in male and female domestic rabbit was 14.11% and 20.34%, respectively. This tendency is consistent with the studies of Tibetan pigs (females, 17.61%; males, 12.72%) [24] and also concurs with that observed in pet parrots (females, 41.22%; males, 30.06%) [14], in wild boars in Germany (females, 83.3%; males, 42.9%) [25] and Italy (females, 45.95%; males, 38.8%) [22]. Females and males were exposed to* Chlamydia* in the same environment, and in our study females are more sensitive to the pathogen than males for each of the breeds in each region group, which may be due to variation in immune response or antibody persistence rates between females and males.

In the present study, there was no significant difference in* Chlamydia* seroprevalence in rabbits in different provinces (*P* > 0.05). The statistically similar seropositivity suggests that the pathogen could be mostly transmitted by the direct contact route in domestic rabbits, which is little affected by environmental change. Thus, further studies are needed to illuminate the potential effect of* Chlamydia* on the geographical origin of domestic rabbits.

*Chlamydia* has been reported to be associated with abortion in horses, rabbits, guinea pigs, mice, and pigs [26]. More importantly,* Chlamydia* can cause devastating consequences of infertility, ectopic pregnancy, chronic pelvic pain, or even abortion in humans [8, 27], especially for people exposed to* Chlamydia* infection by working with rabbits, which increased the possibility to be infected [14, 28]. Thus, further studies are needed to illuminate the potential effect of* Chlamydia* on reproduction of domestic rabbits. Moreover, serological investigation of* Chlamydia* infection in human (particularly pregnant women) working in rabbit farms in northeast China is also warranted.

Among different breeds of domestic rabbits,* Chlamydia* seroprevalence in Chinese White Rabbit (20.88%) was higher than that in New Zealand Rabbit (17.61%) and California Rabbit (15.20%), but the differences were not statistically significant, which was consistent with the previous study, and the animal species factor was calculated and was found to be of no significance [29]. Further investigations should be studied about the correlation of* Chlamydia* infection with the animal species.

In conclusion, the results of the present study revealed that* Chlamydia* infection in domestic rabbits is highly prevalent in northeast China, but this severe situation has been neglected in the past. Therefore, it is important to carry out integrated control strategies and measures to prevent and control* Chlamydia* infection in domestic rabbits in China. This is the first report of* Chlamydia* seroprevalence in domestic rabbits in China.

## Figures and Tables

**Table 1:** Seroprevalence of *Chlamydia* infection in domestic rabbits by age, gender, and breed in China, as detected by indirect haemagglutination assay (IHA).

Factor	Category	Number examined	Number positive	% (95% CI)

Age	Juvenile	464	92	19.83 (16.20–23.46)
	Adult	336	51	15.18 (11.34–19.02)

Gender	Male	319	45	14.11 (10.29–17.93)
	Female	481	98	20.34 (16.78–23.97)

Breed	Chinese White Rabbit	249	52	20.88 (15.84–25.93)
	California Rabbit	250	38	15.20 (10.75–19.65)
	New Zealand Rabbit	301	53	17.61 (13.31–21.91)

Region	Liaoning province	200	39	19.50 (14.01–24.99)
	Jilin province	200	41	20.50 (14.91–26.10)
	Heilongjiang province	200	33	16.50 (11.36–21.64)
	Inner Mongolia Autonomous Region	200	30	15.00 (10.05–19.95)

Total		800	143	17.88 (15.22–20.53)

**Table 2:** Odds ratios for genders of domestic rabbits are taken as risk factors for *Chlamydia* seroprevalence in domestic rabbits.

Gender	Number tested	Number positive	Prevalence (%)	OR (95% CI)	*P* value

Male	319	45	14.11 (10.29–17.93)	Reference	
Female	481	98	20.34 (16.78–23.97)	1.56 (1.06–2.29)	0.024

**Table 3:** Seroprevalence and antibody titers of *Chlamydia* infection in domestic rabbits, as detected by indirect haemagglutination (IHA) test.

Biometric data	Category	Antibody titers							Number positive	Number tested	Prevalence (%)
		1 : 16	1 : 32	1 : 64	1 : 128	1 : 256	1 : 512	1 : 1024			

Gender	Female	17	16	20	18	16	7	4	98	319	20.34
	Male	5	2	13	12	11	2	0	45	481	14.11

Age	Infancy	6	6	8	13	6	2	1	42	175	24.00
	Juvenile	8	6	12	9	10	4	1	50	289	17.30
	Adult	8	6	13	8	11	3	2	51	336	15.18

Region	Liaoning province	5	8	8	10	7	1	0	39	200	19.50
	Jilin province	6	4	8	9	9	3	2	41	200	20.50
	Heilongjiang province	6	4	11	4	6	2	0	33	200	16.50
	Inner Mongolia Autonomous Region	5	2	6	7	5	3	2	30	200	15.00

Breed	Chinese White Rabbit	9	6	14	8	10	4	1	52	249	20.88
	California Rabbit	6	5	8	8	9	1	1	38	250	15.20
	New Zealand Rabbit	7	7	11	14	8	4	2	53	301	17.61

Total		22	18	33	30	27	9	4	143	800	17.88

## References

[1] Longbottom D., Coulter L. J. (2003). Animal chlamydioses and zoonotic implications. *Journal of Comparative Pathology*.

[2] Rohde G., Straube E., Essig A., Reinhold P., Sachse K. (2010). Chlamydial zoonoses. *Deutsches Ärzteblatt International*.

[3] Eidson M. (2002). Psittacosis/avian chlamydiosis. *Journal of the American Veterinary Medical Association*.

[4] Entrican G., Wheelhouse N. M. (2006). Immunity in the female sheep reproductive tract. *Veterinary Research*.

[5] Pannekoek Y., Visser C., Duim B., Heddema E. R. (2009). Chlamydophila psittaci infections in The Netherlands. *Drugs of Today*.

[6] Verminnen K., Vanrompay D. (2009). *Chlamydophila psittaci* infections in Turkeys: overview of economic and zoonotic importance and vaccine development. *Drugs of Today*.

[7] Wu S.-M., Huang S.-Y., Xu M.-J., Zhou D.-H., Song H.-Q., Zhu X.-Q. (2013). *Chlamydia felis* exposure in companion dogs and cats in Lanzhou, China: a public health concern. *BMC Veterinary Research*.

[8] Osman K. M., Ali H. A., Eljakee J. A., Gaafar M. M., Galal H. M. (2012). Antimicrobial susceptibility and molecular typing of multiple *Chlamydiaceae* species isolated from genital infection of women in Egypt. *Microbial Drug Resistance*.

[9] Osman K. M., Ali H. A., ElJakee J. A., Galal H. M. (2012). Chlamydiaceae in riverine buffalo (*Bubalus bubalis*) and cows (*Bos taurus*) in Egypt with and without signs of reproductive disease. *New Zealand Veterinary Journal*.

[10] Beeckman D. S. A., Vanrompay D. C. G. (2009). Zoonotic *Chlamydophila psittaci* infections from a clinical perspective. *Clinical Microbiology and Infection*.

[11] Chen Y. F., Xie X. P., Sun S. K. (2010). The present situation and development countermeasures of Chinese rabbit industry. *Modern Agriculture*.

[12] Boiko E. V., Pozniak A. L., Maltsev D. S., Suetov A. A., Nuralova I. V. (2014). Chronic ocular *Chlamydia trachomatis* infection in rabbits: clinical and histopathological findings in the posterior segment. *Investigative Ophthalmology & Visual Science*.

[13] Chen Q., Gong X., Zheng F., Cao X., Li Z., Zhou J. (2014). Seroprevalence of *Chlamydophila abortus* infection in yaks (*Bos grunniens*) in Qinghai, China. *Tropical Animal Health and Production*.

[14] Zhang N. Z., Zhang X. X., Zhou D. H. (2015). Seroprevalence and genotype of *Chlamydia* in pet parrots in China. *Epidemiology & Infection*.

[15] Tian Y. M., Cao J. F., Zhou D. H. (2014). Seroprevalence and risk factors of *Chlamydia* infection in dogs in Southwestern China. *Acta Tropica*.

[16] Cong W., Huang S. Y., Zhang X. X. (2014). *Chlamydia psittaci* exposure in pet birds. *Journal of Medical Microbiology*.

[17] Stenzel T., Pestka D., Choszcz D. (2014). The prevalence and genetic characterization of *Chlamydia psittaci* from domestic and feral pigeons in Poland and the correlation between infection rate and incidence of *pigeon circovirus*. *Poultry Science*.

[18] di Francesco A., Donati M., Nicoloso S. (2012). Chlamydiosis: Seroepidemiologic survey in a red deer (*Cervus elaphus*) population in Italy. *Journal of Wildlife Diseases*.

[19] Wilson K., Sammin D., Harmeyer S., Nath M., Livingstone M., Longbottom D. (2012). Seroprevalence of chlamydial infection in cattle in Ireland. *The Veterinary Journal*.

[20] Zhou D. H., Zhao F. R., Xia H. Y. (2013). Seroprevalence of chlamydial infection in dairy cattle in Guangzhou, Southern China. *Irish Veterinary Journal*.

[21] Zhang X. X., Li R. C., Liu G. H. (2014). High seroprevalence of *Chlamydia* infection in sows in Hunan province, subtropical China. *Tropical Animal Health and Production*.

[22] di Francesco A., Donati M., Morandi F. (2011). Seroepidemiologic survey for *Chlamydia suis* in wild boar (*Sus scrofa*) populations in Italy. *Journal of Wildlife Diseases*.

[23] Dickx V., Kalmar I. D., Tavernier P., Vanrompay D. (2013). Prevalence and genotype distribution of *Chlamydia psittaci* in feral *Canada geese* (*Branta canadensis*) in Belgium. *Vector-Borne and Zoonotic Diseases*.

[24] Zhang N.-Z., Zhou D.-H., Shi X.-C. (2013). First report of *chlamydiaceae* seroprevalence in Tibetan pigs in Tibet, China. *Vector-Borne and Zoonotic Diseases*.

[25] Hotzel H., Berndt A., Melzer F., Sachse K. (2004). Occurrence of *Chlamydiaceae* spp. in a wild boar (*Sus scrofa L.*) population in Thuringia (Germany). *Veterinary Microbiology*.

[26] Schautteet K., Vanrompay D. (2011). Chlamydiaceae infections in pig. *Veterinary Research*.

[27] Ikeme A. C., Ezegwui H. U., Ikeako L. C., Agbata I., Agbata E. (2011). Seroprevalence of *Chlamydia trachomatis* in Enugu, Nigeria. *Nigerian Journal of Clinical Practice*.

[28] Baud D., Regan L., Greub G. (2008). Emerging role of *Chlamydia* and *Chlamydia*-like organisms in adverse pregnancy outcomes. *Current Opinion in Infectious Diseases*.

[29] Osman K. M., Ali H. A., Eljakee J. A., Galal H. M. (2013). Prevalence of *Chlamydophila psittaci* infections in the eyes of cattle, buffaloes, sheep and goats in contact with a human population. *Transboundary and Emerging Diseases*.

